# Cellular Growth in Aerial Roots Differs From That in Typical Substrate Roots

**DOI:** 10.3389/fpls.2022.894647

**Published:** 2022-05-26

**Authors:** Alen K. Eskov, Violetta A. Viktorova, Evgeny Abakumov, Gerhard Zotz

**Affiliations:** ^1^Faculty of Biology, Lomonosov Moscow State University, Moscow, Russia; ^2^Tzitzin Main Botanical Garden, Russian Academy of Sciences, Moscow, Russia; ^3^Faculty of Biology, Saint Petersburg State University, Saint Petersburg, Russia; ^4^Functional Ecology Group, Institute of Biology and Environmental Sciences, University of Oldenburg, Oldenburg, Germany

**Keywords:** aerial roots, root growth, apical root meristem, epiphytes, hemiepiphytes, nomadic vines

## Abstract

**Background and Aims:**

In the roots of most vascular plants, the growth zone is small, the meristem and the elongation zone are sharply separated, and only meristematic cells divide. This statement is based almost entirely on studies with soil-rooted plants. Whether aerial roots of structurally dependent (=epiphytic/hemiepiphytic) species differ is virtually unexplored.

**Methods:**

Growth of aerial roots in 20 structurally dependent plant species from eight families was studied *ex situ*. In 12 species, we studied the anatomical structure and distribution of cortex cell lengths and rhizoderm in the growth zone.

**Key Results:**

All the studied aerial roots had an open apical meristem, and mitoses were not restricted to the meristem. In contrast to belowground roots, relative growth rate did not strongly increase upon transition to the elongation zone, while elongating growth was often prolonged. Still, the relative growth rate was lower than in belowground roots in soil, and in different species, it did not change considerably compared to each other.

**Conclusions:**

A distinct elongation zone with rapid cell growth was missing in the studied aerial roots. Rather, there was a growth zone in which division, growth, and differentiation co-occurred. We observed a generally low relative growth rate in aerial roots and a surprisingly similar initial growth pattern in spite of the diversity in taxonomy and ecology, which resembled initial cellular growth in leaves, stems, and fleshy dicotyledonous fruit.

## Introduction

Sachs (1887), by direct observation, was apparently the first who have described three stages of plant organ growth: (1) meristematic, during which new cells are formed and separated into tissues; (2) elongation, with rapid cell growth until organs reach their final size; and (3) maturation, when final features of different cell types develop. The vast majority of cellular root growth studies have been performed on a limited number of species, mainly the roots of crop seedlings and *Arabidopsis thaliana*, which have long been the primary models for the study of the root apical meristem (RAM), its organization, ontogenesis, and growth regulation mechanisms. The apical meristem of such roots is characterized by active proliferation, laying of cell rows with symplastic growth, and rapid change in cellular organization. The Sachs descriptions are applicable to some cells and organs, but not for all of them. Nowadays, the distribution of growth in the root is considered as a most effective system for study than growth in other plant organs, and the root is characterized by strictly columnar growth of all cells in the meristem (Evert, 2006).

Data on root growth rates are relatively scarce in the literature. As a rule, pertinent studies utilized a rhizotron and on agricultural crops juvenile plants (Cahn et al., 1989). Currently, the largest study on root growth compared 73 flowering plant species, including only ruderal geophyte seedlings and crops (Zhukovskaya et al., 2018).

The root apical meristem (RAM) is located in the growing tip of the main, subordinate, or lateral root. Based on cell proliferation direction, there is a closed RAM or an open RAM (Clowes, 1981, 1982; Groot et al., 2004; Evert, 2006), reflecting the presence or absence of a clear anatomical boundary between root cap and root proper. The open type is probably ancestral among angiosperms, but the closed type is most common in extant species (Heimsch and Seago, 2008). In some species, the meristem decreases in size and changes from a closed type to an open type during ontogenesis (Armstrong and Heimsch, 1976; Clowes and Wadekar, 1989) or disappears over time.

We hypothesized a fundamentally different structure and nature of growth in aerial roots because these do not experience substrate root-soil resistance (Eskov et al., 2016). Even at the dawn of experimental biology, aerial root growth was observed to be different from that of the substrate roots (Went, 1895; Linsbauer, 1907; Blaauw, 1912). It has been claimed for some species that the growth zone of aerial roots can reach many tens of centimeters (Jost, 1908), which suggests a different organization of growth processes from subterranean roots. Although relatively less is known about aerial root growth, there are some studies on the anatomy and morphology of aerial roots in general and aerial roots of epiphytes in particular. Considerable anatomical differences in aerial roots from belowground ones have been observed in mangroves in the family Rhizophoraceae, in which cell divisions in aerial roots were observed over a length of up to 23 cm. These roots also contained chlorophyll, trichosclereids (long fiber cells with lignified walls), which are more typically found in stems (Gill and Tomlinson, 1971). Once aerial roots reach the substrate, lateral roots develop, with a very different structure from that of aerial ones: trichosclereids are not present, chlorophyll is absent, and the differentiation of protoxylem begins after the short elongation zone (Gill and Tomlinson, 1977).

The presence of trichosclereids is also the characteristic for aerial roots, in the cortex of which, they are located in the intercellular spaces of the climbing *Monstera deliciosa* (Bloch, 1944, 1946; Sinnott and Bloch, 1946). Hinchee (1981, 1983) discussed the anatomical data in the context of growth and continued the study of trichosclereids and idioblasts in M. deliciosa roots started by R. Bloch, comparing their growth and structure during the transition from aerial to subterranean growth. Growth processes of aerial roots of epiphytes and hemiepiphytes have rarely been studied, although there has been extensive research devoted to root anatomical and morphological features, as reviewed in Gill and Tomlinson (1975) and Benzing (1996).

Although the origin and development of aerial roots are rarely discussed by itself, as aerial roots are almost always adventitious, parallels can be drawn when discussing their origin. Barlow (1994) distinguishes adventitious and “shoot-born” roots. The former originates, in the broadest sense, in the root pole of the embryo, the latter can grow from a stem or leaf and, in some cases, is the regenerative response (Barlow, 1994). The aerial roots also exhibit anatomical structure characteristic of the stems, for example, the aforementioned trichosclereids (Bloch, 1946; Gill and Tomlinson, 1971), or photosynthetic activity, as in leaves and some stems.

Thus, there is much indirect and scattered evidence that allows us to consider alternative root growth patterns, but to date, a comprehensive analysis of growth in aerial roots has not been conducted. Thus, the aim of our work was to study aerial root growth of structurally dependent angiosperms, i.e., epiphytes, hemiepiphytes, and nomadic vines (see Zotz et al., 2021). We have explored the following questions: (1) whether the structure of RAM and the growth pattern of aerial roots differ significantly from the belowground roots of traditional model species? (2) If the growth of aerial root similar to the growing of other axial organs? (3) Do aerial roots lack a clearly localized border between the RAM and the differentiation zone?

## Materials and Methods

Experimental plants were cultivated in the Main Botanical Garden greenhouses of the Russian Academy of Sciences (Moscow) under microclimatic conditions similar to humid tropical forest conditions (Table 1). The aerial roots (without contact to a substrate) of 20 species of epiphytes, hemiepiphytes, and nomadic vines from representatives of eight families were sampled (Table 2). Observations were carried out during summer period of active growth. Watering and maintaining air humidity was carried out with water purified by reverse osmosis (i.e., close to rain). The average monthly photosynthetically active radiation varied in the summer period within 200–259 MJ/m^2^, relative humidity from 75 to 95%. Air temperature varied during the summer experiment from 29 (maximum daily day) to 18°C (minimum night time). When the temperature dropped below 18°C, heating was turned on. All plants were cultivated in suspended form or in baskets (without soil). The number of plants available for the study ranged from 2 to 6 per species (for details refer to Table 2) and was growing side by side. Each repetition included 20 roots. A number of three repetitions were used for each measurement. Thus, a total of 60 roots of each species were investigated. First repetition was done in early June, second repetition at end of June, and the third one in early or mid-July.

**Table 1:** Microclimatic parameters of the tropical greenhouse Tzitzin Main Botanical Garden where the experiments were conducted.

**Parameters**	**Winter**	**Spring**	**Summer**	**Autumn**
Daily temperature	20–22°C	22–27°C	24–29°C	21–25°C
Night temperature	18–20°C	18–21°C	18–23°C	18–22°C
^*^Monthly sums of solar irradiance, MJ/m^2^.	34–122	285–590	468–604	54–311
^*^Monthly sums of photosynthetically active radiation, MJ/m^2^	13–47	111–249	200–259	21–125
^**^Relative humidity	60–75%	75–95%	75–95%	65–80%

* *For Moscow, based on many years of observations of the beginning of the twenty-first century, excluding the anomalous 2010. Source: Lomonosov University*.

** *Relative humidity is lower in the cold season due to greenhouse heating*.

**Table 2:** A summary table of the parameters characterizing the root growth of the studied flower epiphytes species *ex-situ* (mean ± 1SD).

Family (^*^)	Species (Ecobiomorph^**^)	***n***	**Root growth parameters**				
			**D, mm**	**L_**p**_, mm**	**V, mm/d**	**k_**p, **_ d^**−1**^**	**L_**p**_/D**
Orchidaceae (66.88%)	*Cattleya skinneri* (S.l.e.)	3	2.9 ± 0.2	2.2 ± 0.9	2.1 ± 0.6	0.33	0.7
	*Cymbidium finlaysonianum* (N.e.)	3	1.1 ± 0.2	1.8 ± 0.6	0.8 ± 0.3	0.15	1.6
	*Grammatophyllum speciosum* (N.e.)	2	1.5 ± 0.4	1.5 ± 0.5	0.5 ± 0.2	0.13	1.1
	*Microcoelia exilis* (R.ph.l.)	3	1.1 ± 0.2	1.6 ± 0.5	0.6 ± 0.3	0.15	1.6
	*Neofinetia falcata* (S.l.e.)	3	2.6 ± 0.5	2.6 ± 1.2	0.8 ± 0.2	0.15	1.2
	*Stanhopea tigrina* (N.e.)	3	2.5 ± 0.5	1.3 ± 0.1	0.7 ± 0.0	0.16	0.7
Araceae (6.46%)	*Anthurium crassinervium* (A.e.)	3	3.6 ± 0.5	3.1 ± 0.7	1.4 ± 0.7	0.12	0.8
	*Anthurium gracile* (A.e.)	4	3.4 ± 0.6	2.1 ± 0.2	0.6 ± 0.2	0.15	0.6
	*Monstera deliciosa* (N.v.)	6	5.3 ± 1.4	25 ± 7.1	9.6 ± 5.3	0.42	4.3
	*Philodendron scandens* (N.v.)	3	1.5 ± 0.8	15 ± 4	4.4 ± 2.3	0.22	10.1
Bromeliaceae (5.48%)	*Hohenbergia penduliflora* (R.e.)	4	1.2 ± 0.2	1.1 ± 0.2	1.5 ± 0.5	0.32	0.9
	*Neoregelia carolinae* (R.e.)	4	0.8 ± 0.2	0.5 ± 0.1	1.4 ± 0.6	0.57	0.6
	*Tillandsia ionantha* (A.e.)	5	0.9 ± 0.2	2.2 ± 0.7	0.5 ± 0.1	0.09	2.2
Moraceae (2.5%)	*Ficus formosana* (Str.)	2	1.1 ± 0.2	4.3 ± 0.7	10.9 ± 5	1.09	3.8
Rubiaceae (1.06%)	*Hydnophytum formicarum* (S.m.)	5	0.7 ± 0.3	1.2 ± 0.4	0.6 ± 0.2	0.15	1.6
	*Myrmecodia tuberose* (S.m.)	3	1.1 ± 0.2	1.5 ± 0.6	0.7 ± 0.3	0.15	1.3
Cactaceae (0.71%)	*Rhipsalis mesembryanthemoides* (S.s.)	4	0.5 ± 0.1	1 ± 0.1	0.1 ± 0.1	0.10	1.0
Apocynaceae (0.63%)	*Dischidia nummularia* (E.v.)	3	0.5 ± 0.1	0.7 ± 0.3	0.2 ± 0.1	0.11	1.2
	*Hoya carnosa* (E.v.)	5	1 ± 0.3	1.2 ± 0.6	0.4 ± 0.1	0.13	1.2
Zingiberaceae (0.09%)	*Hedychium bousigonianum* (N.e.)	2	6 ± 0.9	25 ± 7.3	5.1 ± 1.7	0.11	4.2

*Parameters are D = root thickness (diameter) in mm; (Lp) growth zone length in mm; (V) average growth rate in mm/d; (kp) relative growth rate in h^−1^. n, number of replicate plants (families are located on the scale of biodiversity of each group among epiphytes)*.

* *Family and its participation in epiphyte biodiversity, % (after Benzing, 1990)*.

** *Ecobiomorph: A.e., “Atmospheric” epiphytes; N.e., “Nesting” epiphytes; Str., Stranglers; R.e., “Reservoir” epiphytes; R.ph.l., Root photosynthesizing, leafless; S.l.e., Succulent leaf epiphytes; N.v., nomadic vines; S.m., stem myrmecophiles; S.s., stem succulents; E.v., epiphytic vines*.

### Growth Rate Measurement

Along the root length, ink marks were applied to the growing area with a thin brush at intervals of 0.5–2 mm (the mark dimensions depended on root thickness). The first mark was applied to the root tip. The growth rate was measured with a millimeter ruler at different times depending on the rate, but in most species, the interval was at least 5 days.

**Figure 1:**
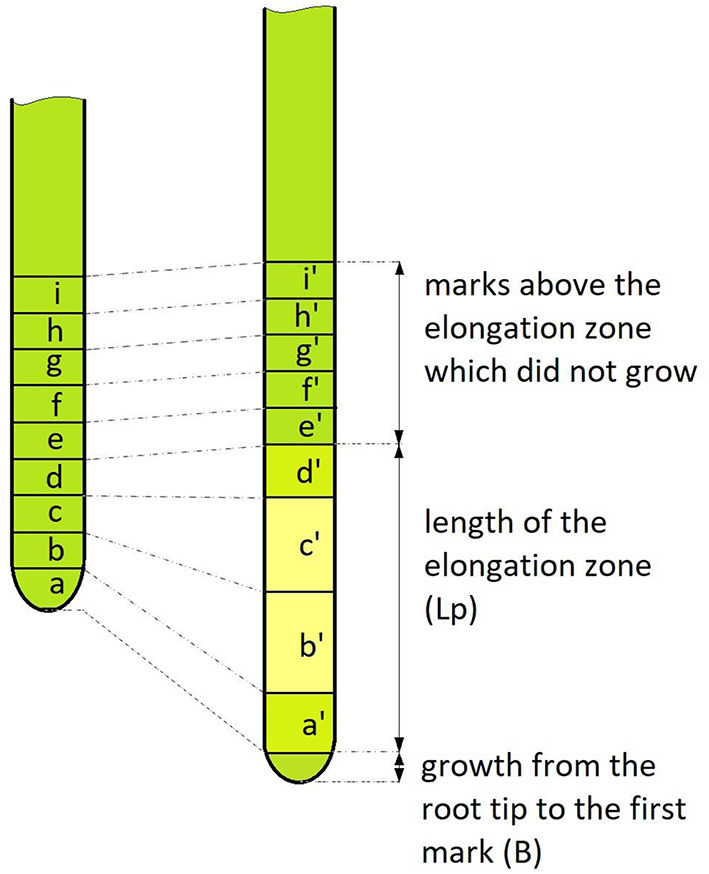
Schematic picture of the applied growth rate measurement model. On the left is the root before the start of the measurement, on the right—after. A number of uniform marks were applied to the root (nine in this example). The marked zone after the measurement period (T, which mainly lasted 5 or 10 days) shows that some of the marks grew (marks a, b, c, d), forming the elongation zone Lp. Also, the root grew from the last mark (B), which was not observed in all dimensions. The ratio of the length of the growth zone after and before the start of observation gave the growth coefficient S = (a' + b' + c'+ d')/(a + b + c + d). Thus, formula (1) by which we calculated the growth rate is equivalent to the net increase [(a' + b' + c'+ d') – (a + b + c + d) + B] divided by the measurement interval (T).

The average growth rate of the entire root (V) (in mm/day) was calculated using a previously published formula (Eskov et al., 2016, refer to Figure 1):


(1) $$\begin{array}{r} V=\left(L_{\text{p}}S+B-L_{\text{p}}\right)/T_{,} \end{array}$$

in which S is the ratio of the total length of elongation zone after and before the start of observations (growth coefficient), L_p_ is the length of the elongation zone at the start of the observation, B is the growth from the root tip to the first mark, and T is the interval between measurements. The relative growth rate (k_p_) per day (d^−1^) was calculated using the following formula (Evans, 1972):


(2) $$\begin{array}{r} k_{\text{p}}=\left(1/t\right)\;ln\left(Lf/Li\right) \end{array}$$

where Δt is time, Lf is final length, Li is initial length, and the ratio Lf/Li is identical to the coefficient S from the previous formula.

### Anatomical Structure

The aerial root growth zone was studied on permanent microscope slides of individuals from 12 species (Table 3). The sampling of roots was done from the same experimental plants and at the same time when the rate of root growth was measured, i.e., in summer (June–July). The roots were fixed in a mixture of formalin: 96% ethanol: glacial acetic acid (9.5: 2.5: 2, v/v/v). Thick roots were left in the fixing agent for 24 h, thin roots for 1.5–2 h. Before mounting in paraffin, the time of stay at each alcohol portion was extended to 12 h. Paraffin was dissolved in chloroform and butanol. To cover the elongation zone as much as possible, large roots were cut into pieces from 3 to 7 cm. Tissues were sliced using a BASE SLEDGE microtome (MSE, United Kingdom) to produce sections between 10 and 20 μm thickness. The sections were stained according to Feulgen with subsequent tinting with 0.1% alcian blue 8GS in 3% acetic acid for 30 min at room temperature. A number of slides were stained using the PAS method with subsequent tinting with 0.15% solution of Procion Brilliant Blue 4RS in 0.2% solution of Na_2_CO_3_ during 30–40 min at room temperature. These methods ensure good staining of cell walls and surrounding slime. After staining, sections were mounted in Canada balsam. The slides were examined with the CX41 microscope (Olympus, Japan).

**Table 3:** Parameters characterizing the cortical and rhizodermal growing zone cells growth of 12 species from the families Orchidaceae, Araceae, Bromeliaceae, Moraceae, Cactaceae, obtained by analysis of longitudinal root sections.

			**Slicer parameters**				
**Family**	**Species (Ecobiomorph)**	***n***	**Growth zone boundary, mm**	**Cortical cell length, μm**	**Rhizodermal cell length, μm**	**Cell formation rate, cortex** **(cells/h)**	**Cell formation rate, rhizoderm (cells/h)**
Orchidaceae	*Cattleya skinneri* (S.l.e.)	4	4.1 ± 0.2	122 ± 14	71 ± 11	0.7 ± 0.1	1.2 ± 0.2
	*Cymbidium finlaysonianum* (N.e.)	5	4.3 ± 0.3	316 ± 13	152 ± 12	0.1 ± 0.1	0.2 ± 0.1
	*Grammatophyllum speciosum* (N.e.)	8	2.7 ± 0.4	107 ± 16	44 ± 2	0.2 ± 0.1	0.5 ± 0.1
	*Neofinetia falcata* (S.l.e.)	9	5.8 ± 0.2	199 ± 17	49 ± 3	0.2 ± 0.1	0.7 ± 0.1
	*Stanhopea tigrina* (N.e.)	5	3.1 ± 0.2	103 ± 10	61 ± 5	0.3 ± 0.1	0.5 ± 0.1
Araceae	*Anthurium crassinervium* (A.e.)	5	8.0 ± 0.7	121 ± 13	43 ± 5	0.5 ± 0.1	1.4 ± 0.5
	*Anthurium gracile* (A.e.)	10	5.4 ± 0.5	167 ± 9	107 ± 11	0.1 ± 0.1	0.21 ± 0.1
	*Monstera deliciosa* (N.v.)	10	25 ± 2.2	308 ± 9	103 ± 10	1.3 ± 0.1	3.9 ± 0.4
	*Philodendron scandens* (N.v.)	12	6.1 ± 0.2	109 ± 20	46 ± 7	1.6 ± 0.3	3.7 ± 0.6
Bromeliaceae	*Tillandsia ionantha* (A.e.)	5	2.2 ± 0.2	99 ± 5	57 ± 4	0.2 ± 0.1	0.4 ± 0.1
Moraceae	*Ficus formosana* (Str.)	8	4.7 ± 0.5	177 ± 10	43 ± 4	2.4 ± 0.3	10 ± 1
Cactaceae	*Rhipsalis mesembryanthemoides* (S.s.)	5	2.2 ± 0.3	75 ± 8	50 ± 5	0.1 ± 0.1	0.1 ± 0.1

*Data are means ± SD*.

Microphotographs were taken using an Altra20 camera (Olympus, Japan). Rhizoderm and cortex cell length were measured in c. 2,000 micrographs using the ImageJ program. These data were used to plot growth distribution over the elongation zone of all 12 species and to obtain parameters characterizing cell growth. Cell length was maximal behind the boundary of the growth zone, near which there was clearly no more increment in cell length. Cell formation rate (per hour) was determined by dividing root growth rate (V, μm/h) by the cell length that completed growth. In intensively growing roots of hemiepiphytes (*Monstera deliciosa, Philodendron scandens*, and *Ficus formosana*), the division termination location was determined visually, in slowly growing roots of typical epiphytes, it was after the termination of a periodic decrease in the average cell length. On the graphs, the maximum value among the studied slices is taken as the end of division and is indicated by a red marker on the finally prepared average graph.

### Statistical Analysis

Statistical analysis was performed in Microsoft Excel 2007 to determine the averages and standard errors. To evaluate the relationships between the various studied parameters characterizing root growth, we used correlation analyses, calculating the non-parametric Spearman's correlation coefficient in STATISTICA 12.0, with *p* < 0.05 as the critical value for significance. The calculations of the growing root cell length distributions within individual species and the determination coefficients to the linear approximations of the graphs were performed in Excel 2007.

## Results

The average aerial root in orchids growth rate was low (0.5–1.9 mm/d), and the relative rate was approximately constant (0.13–0.33 d^−1^) (Table 2; Supplementary Table S1). In aroids, the average root growth rate ranged from 0.5 mm/d in *Anthurium gracile* to 9.6 mm/d in *Monstera deliciosa*. The elongation zone length varied depending on root thickness. In *Monstera*, this zone was c. 3 cm in the thinnest and ≥ 10 cm in the thickest roots. Both *Monstera deliciosa* and *Philodendron scandens* had long elongation zones and high growth rates, whereas nest epiphytes *Anthurium crassinervium* and *Anthurium gracile* had shorter zones and lower growth rate. However, the relative speed for all four species was very similar (c. 0.22 d^−1^).

Root growth rates were highly variable among the representatives of bromeliads. A high relative root growth rate was found in the tank bromeliad in *Neoregelia carolinae*, the maximum value of 0.57 d^−1^ being the highest of all the studied species bromeliads at a low average root growth rate, whereas *Tillandsia ionantha*, an atmospheric epiphyte, featured slow low average and relative root growth (Table 2).

The aerial roots of the remaining species also grew with low average and relative growth rates (0.1–5 mm/d and 0.10–0.15 d^−1^, respectively) (Table 2). The highest average and relative growth rate among these species were of the hemiepiphyte-strangler *Ficus formosana* (11 mm/d and 1.09 d^−1^, respectively): they reach the ground as soon as possible, after which, a second thickening and coalescence begin, leading to “strangulation” of the host.

**Figure 2:**
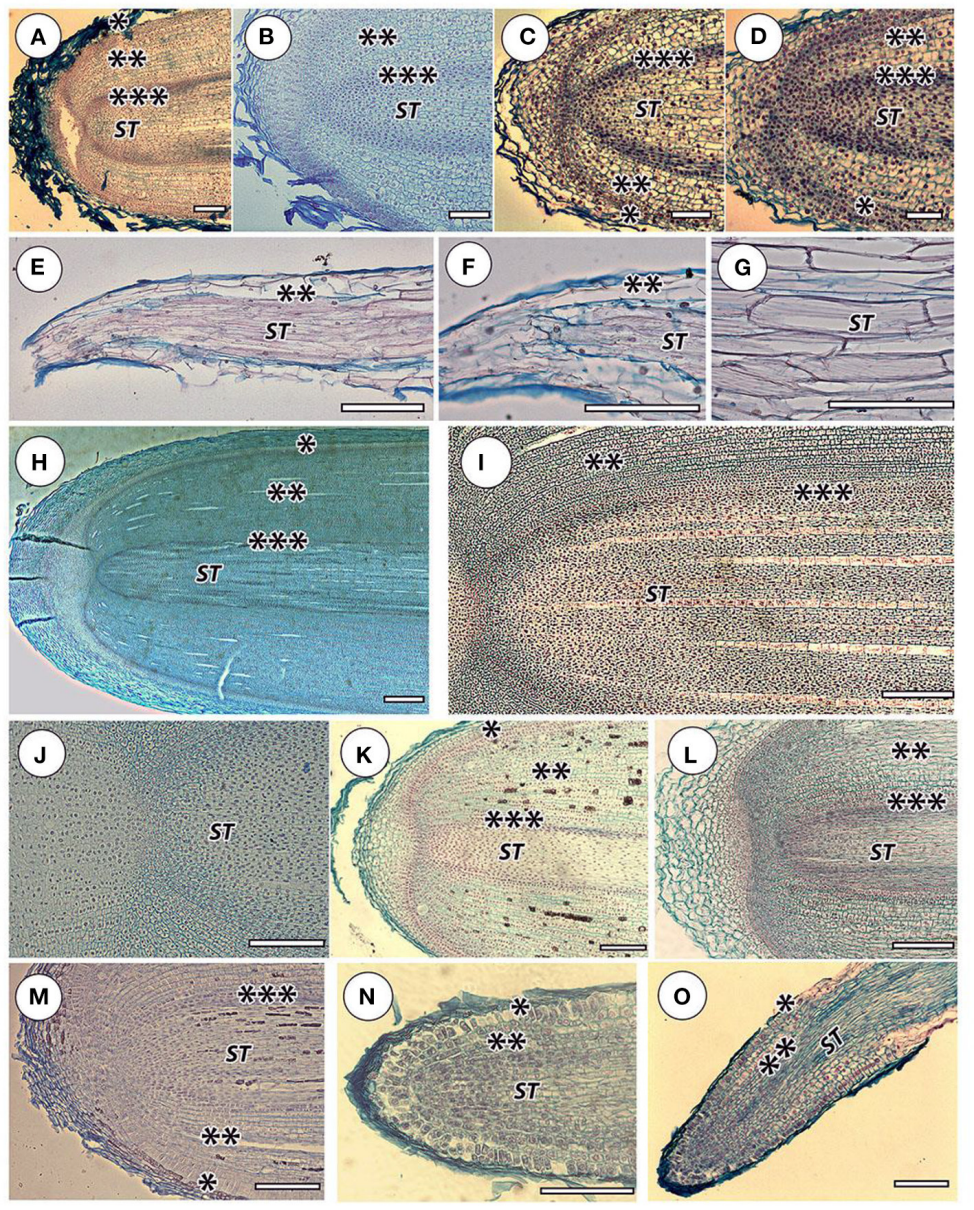
Apical zones in selected species. Orchidaceae: **(A)** *Cattleya skinneri*, **(B)** *Neofinetia falcata*, **(C)** *Cymbidium finlaysonianum*, **(D)** *Grammatophyllum speciosum*; *Stanhopea tigrine*. Lateral root catchers: **(E)** general root view, longitudinal section, **(F)** apical root part, clear that the meristem is absent, **(G)** stele cells in the middle root part, it is clear that the walls are fibrous and thickened. Araceae: **(H)** *Monstera deliciosa*—general view of the thick root apical meristem, **(I)** *M. deliciosa*—general view of the thin root apical meristem, **(J)** *M. deliciosa—*central meristem part, **(K)** *Anthurium crassinervium*. Bromeliaceae: **(L)** *Tillandsia ionantha*. Moraceae: **(M)** *Ficus formosana*. Cactaceae: **(N)** *Rhipsalis mesembryanthemoides*—apical part, **(O)** *Rhipsalis mesembryanthemoides—*elongation zone, general view. Sliced tissue: (*) protoderm and/or rhizoderm (in orchid velamen); (**) cortex; (***) pericycle; *(ST)* stele. Scale 100 μm; except (H)-−1,000 μm.

The root apical meristem (RAM) in the studied orchid species was open: the meristem rose high along the pericycle and protoderm (Figures 2A–D). In this sense, orchids and other studied groups are not fundamentally different in terms of RAM structure. We have discovered a special type of anatomical structure in ageotropic lateral catcher roots in nesting epiphyte orchids. Lateral ageotropic roots of Grammatophyllum speciosum and Stanhopea tigrina have no meristem in the adult state (Figures 2E–G). It can be assumed that RAM is initiated at the earliest stage of the formation of the primordia of the catcher root from the cells of the pericycle, but is exhausted as it grows, and after elongation by stretching of all the embedded cells, growth stops.

**Figure 3:**
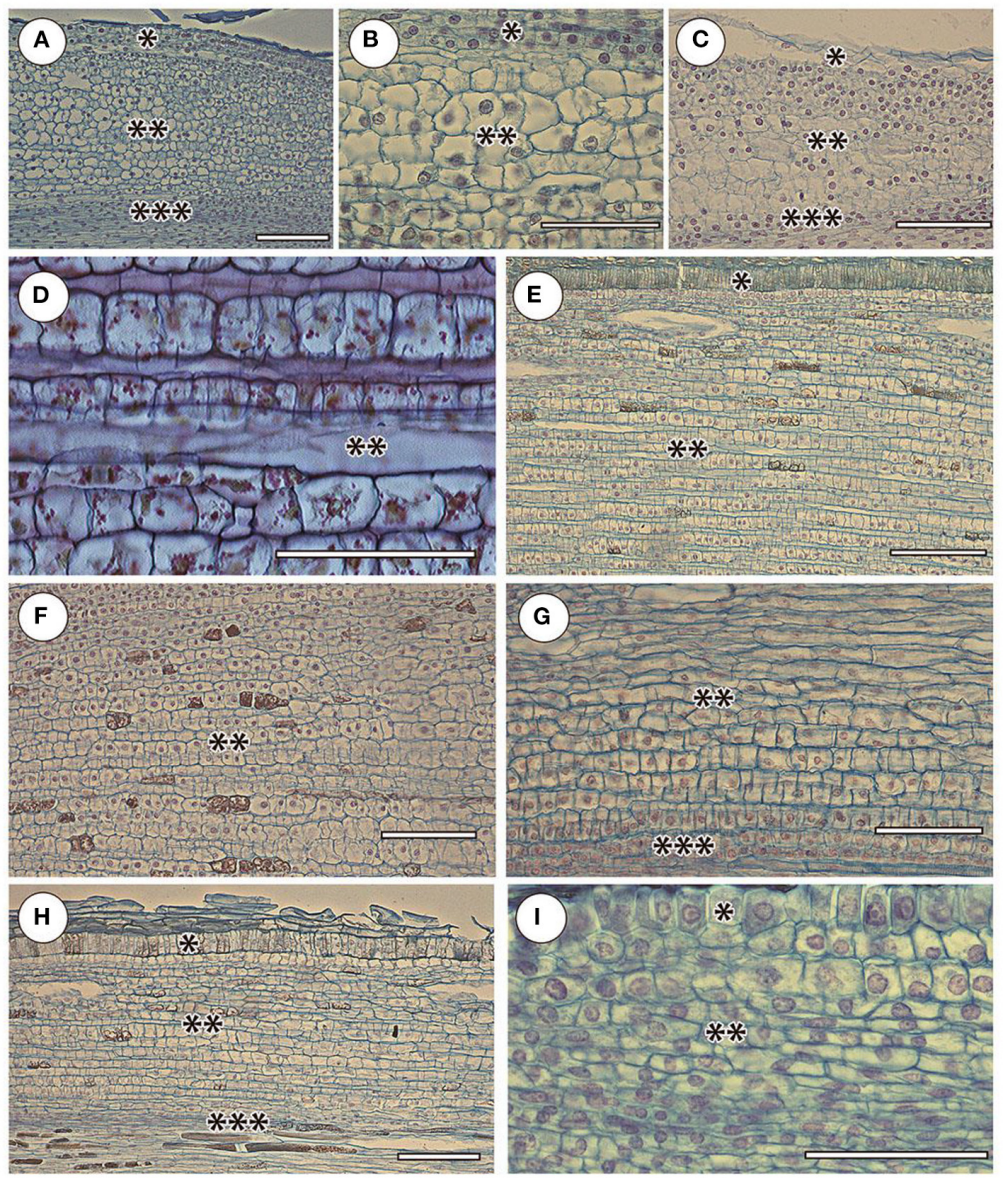
Cortex and adjacent tissues in selected species in the elongation zone. Orchidaceae: **(A)** *Cattleyas skinneri*, **(B)** *Neofinetia falcata*, **(C)** *Cymbidium finlaysonianum*. Araceae: **(D)** *Monstera deliciosa*, **(E)** *Philodendron scandens*, **(F)** *Anthurium crassinervium*. Bromeliaceae: **(G)** *Tillandsia ionantha*. Moraceae: **(H)** *Ficus formosana*. Cactaceae: **(I)** *Rhipsalis mesembryanthemoides*. Sliced tissues: (^*^) protoderm and/or rhizoderm (in orchid velamen); (^**^) cortex; (^***^) pericycle. Scale 100 μm.

The cortex of orchid roots was not folded by smooth cell rows, but by curved cell packets (polycytes) (Figures 3A–C); however, their development is less pronounced than in aroids. Thus, between them, there was no well-developed network of intercellular spaces characteristic of aroids (Figures 3D–F). In the aroids, all the typical features of aerial roots were essentially pronounced (Figures 2H–K). Specific features were the presence of idioblast and lactic. The development of polycytes was most pronounced (Figures 3D,E).

The anatomy of the bromeliads (e.g., *Tillandsia ionantha*) was similar to that of other aerial roots. The RAM was open (Figure 2L), the cortex was composed of polycytes morphologically similar to the rest of the studied species (Figure 3G), and the rhizoderm in many ways resembled orchid velamen (the presence of velamen in the bromeliads has been repeatedly discussed, e.g., Kowalski et al., 2019).

The RAM of other families and species of open or mixed type was not clearly differentiated by primary tissues (Figures 2M–O). The root cap is more or less reduced. The periblem and cortex have a polycyte structure (Figures 3H,I). In the thinnest roots (*Rhipsalis mesembryanthemoides*, Table 2), the separation of the central root cylinder into stele and cortex in the elongation zone was very implicit (Figures 2N,O).

The distribution of cortical and rhizodermal cell growth showed several trends. The boundary of the growth zone normally exceeded the length of the elongation zone of the growing root (Table 3). Rhizodermal cells were always shorter than cortical cells, ranging from 43 ± 5 μm in *Anthurium crassinervium* to 152 ± 12 μm in *Cymbidium finlaysonianum*. The cortical cell length varied 5-folds from 74 μm in *Rhipsalis mesembryanthemoides* to 316 μm in *Cymbidium finlaysonianum* (Table 3). The cell formation rate (cells/hour) was highly variable, from <1 cell in 10 h in the *Rhipsalis mesembryanthemoides* rhizoderm to 2.4 ± 0.3 in the cortex and 10.0 ± 1.0 in the *Ficus formosana* rhizoderm (Table 3). In general, <1 cell/h was typical for the majority of studied species. The cell formation rate in the rhizoderm was higher than in the cortex, both by species and on average (Table 3), which validate the high proliferation activity in the rhizoderm also observed by other methods (see below).

**Figure 4:**
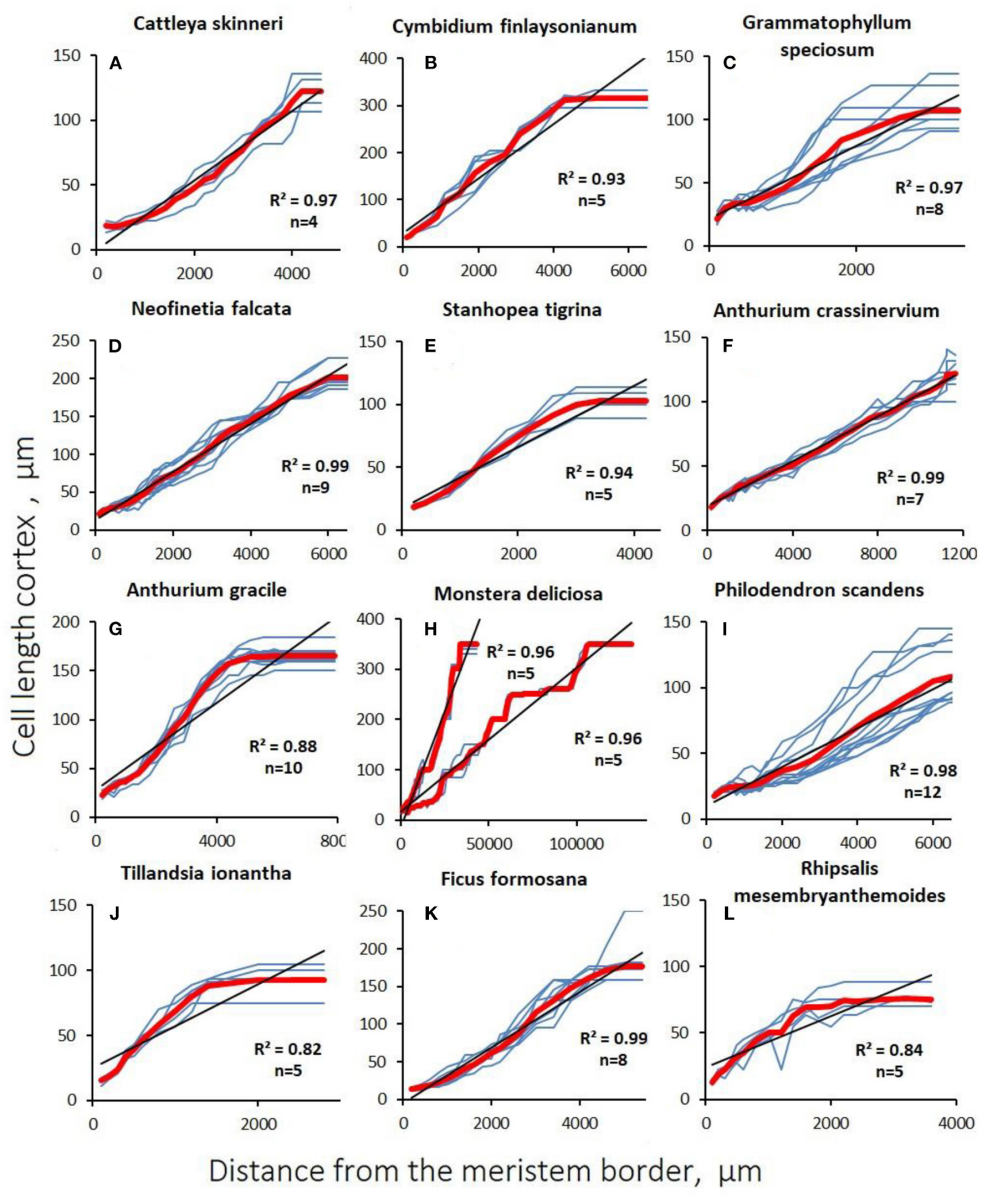
Changes in cortex cell length with distance from the apex (distal apical meristem border) in aerial roots. Blue lines are data for a single studied root section of the root. The red line is the average value. The coefficient of determination is given to the average value, (*n*) is the number of sections examined. **(A–E)** Orchidaceae; **(F–I)** Araceae; **(J)** Bromeliaceae; **(K)** Moraceae; **(L)** Cactaceae.

**Figure 5:**
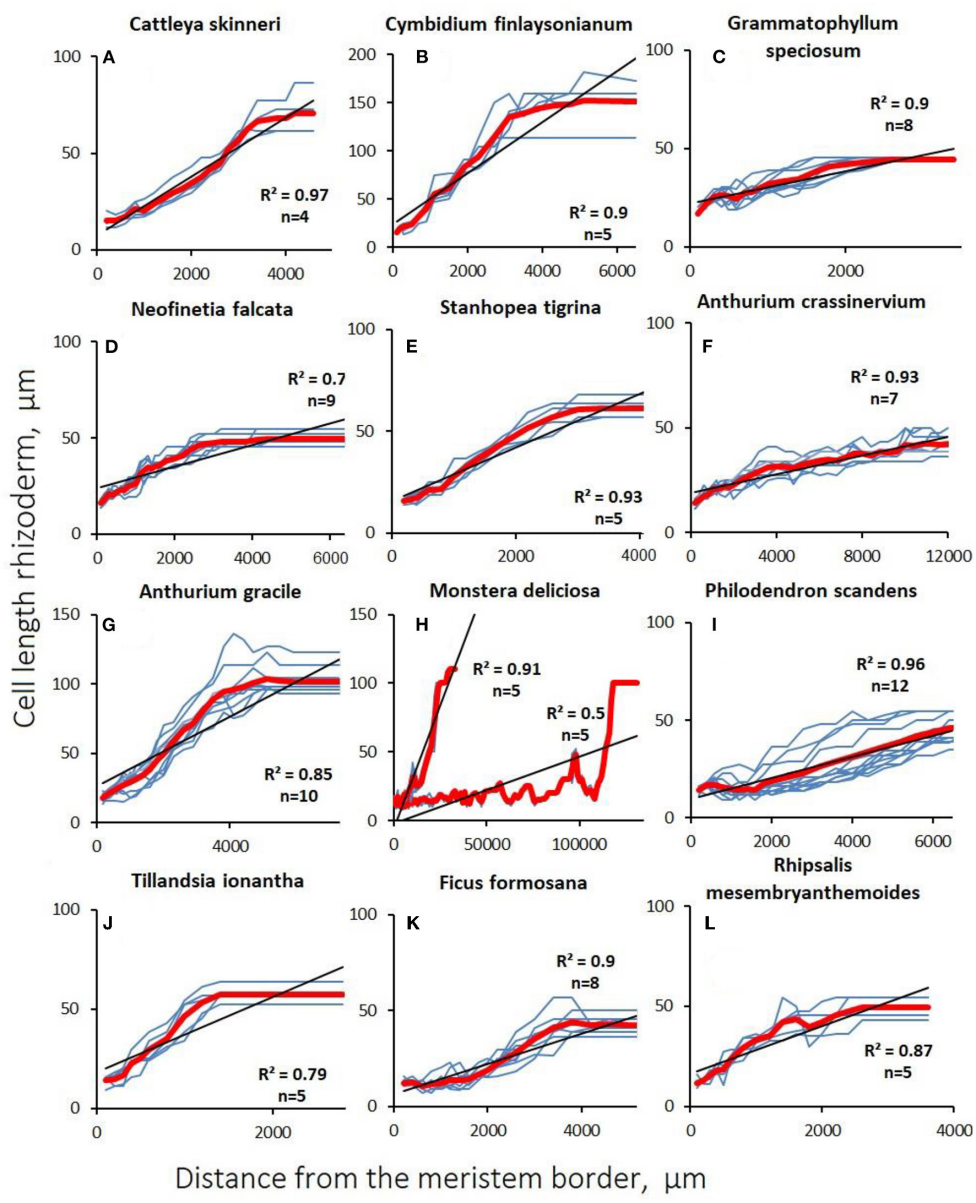
Changes in rhizodermal cell length with distance from the apex (distal apical meristem border) of aerial roots. Gray lines are data for a single studied root section of the root. The red line is the average value. The coefficient of determination are given to the average value, (*n*) is the number of sections examined. **(A–E)** Orchidaceae; **(F–I)** Araceae; **(J)** Bromeliaceae; **(K)** Moraceae; **(L)** Cactaceae.

The graphs of the linear cortical and rhizodermal cell growth show the presence of numerous “steps and dips” that indicate the presence of dividing cells deep in the growth zone (Figures 4, 5; Supplementary Table S2). With a finite cell length of both cortex and rhizoderm of, on average, 100–150 μm, the root growth rate was equivalent to an increase of <1 cell per row per hour in most species. Similar to rapidly growing hemiepiphytic roots, the rate of cell formation in the epiphyte cortex ranged from 0.06 ± 0.01 to 0.67 ± 0.08 cells/h (Table 3).

In aerial roots, there was no sharp increase in cell growth after cessation of division in the cortex. In contrast to substrate roots, the growth of cortical (Figure 4) and rhizodermal cells (Figure 5) was always different, and the slope of the linear approximation graph which characterizes the correlation of cell length with distance from the tip was lower in the rhizoderm than in the cortex, which is explained by the later division termination in the rhizoderm than in the cortex (Figures 4, 5).

## Discussion

We showed that the aerial root growth rate is substantially lower than that of substrate roots. This applies to both absolute and relative values. Substrate roots are generally characterized by high average and relative growth rates. For example, for corn grown in a rhizotron, the root growth rate is 3 cm/d (Cahn et al., 1989). For a cucumber, it is 1.1 mm/h, which is comparable to the hypocotyl growth rate of 1 mm/h (Shirokova and Ivanov, 1997), or leaf of 2.3 mm/h (Kavanová et al., 2006). The few data on the growth of aerial roots in nature are contradictory. The difference in average growth rates between hemiepiphytic Aroids *Philodendron radiatum* (29.6 mm/d) and *Anthurium clavigerum* (9.6 mm/d) was more than three-fold. Only for *A. clavigerum*, there was a significant effect of season on root growth. In this species, aerial roots grew two times as fast in the rainy (12.5 mm/d) than in the dry season (6.3 mm/d) (Meyer and Zotz, 2004).

A comparison of the relative root growth rates (in h^−1^) more accurately reflects the real cell growth rate in the elongation zone: for cucumber, it is 0.38 (Shirokova and Ivanov, 1997), for timothy grass, 1.27, for corn, 0.4, and horse beans, 0.1 (Silk, 1984). For other vegetative organs, the relative growth rates are much lower: ryegrass leaf is 0.064 (Kavanová et al., 2006), rough cocklebur, 0.013, cucumber, 0.01, grape, 0.004 (Silk, 1984); cocklebur apex shoot, 0.009, buttercup, 0.006 (Silk, 1984); and the cucumber hypocotyl, 0.06 (Shirokova and Ivanov, 1997). Thus, the relative root growth rate, as a rule, is an order of magnitude higher than that of the other plant vegetative organs, although their average growth rate is comparable (Ivanov, 2011). The length of root cells that completed growth in the substrate is quite large (1 mm) (Silk et al., 1989); in general, these roots are characterized by an acceleration of root growth rate in proportion to the increase in dividing cells under ontogenesis (Beemster and Baskin, 1998). As our data show, the relative growth rate of aerial roots is closer to the growth of other vegetative organs than roots. This is due to lower absolute velocities with a longer growth zone. The length of the meristem in belowground roots in both monocotyledonous and dicotyledonous taxa correlates positively with root thickness (Bystrova et al., 2018). We observed the same trends for aerial roots.

Cells that have completed growth in aerial roots were much smaller than those which grew in substrate (Silk et al., 1989; Ivanov, 2011). In roots of seedlings of agricultural crops and *Arabidopsis*, the boundary between meristem and extension zone is clearly visible, with a sharper change in average cell length with increasing distance from the tip (Ivanov and Dubrovsky, 2013). In aerial roots, there is no such sharp change in average cell length, neither in the cortex nor in the rhizoderm. In the cortex, most mitoses are concentrated in the root apical portion; however, in some cases, they spread much further. Features of the cellular organization growth differ significantly between aerial and belowground roots. In most studied roots, the growth zone is small, the meristem and the extension zone are sharply separated, and only meristematic cells divide (Bystrova et al., 2018; Zhukovskaya et al., 2018). The relative growth rate is high, and in meristematic cells, it is even higher than in most elongated cells of other organs. In growing root cells, the relative growth rate is several times higher than in meristematic cells. Mitotic cycles in most species last 10–15 h, and cell stretching lasts for 6–15 h (Zhukovskaya et al., 2018). Cells remain in the meristem for no more than a few days. Thus, the roots are characterized by a rapid change of growing cells, and long-term maintenance of growth is due to initial cell divisions. In other vegetative organs, the meristem passage and the extension zone by the cells are much longer than in the roots. In different organs, there are the differences in the organization of growth at the cellular level, but these features of the root organization and vegetative organ growth at the cellular level can be clearly identified (Ivanov, 2011).

**Figure 6:**
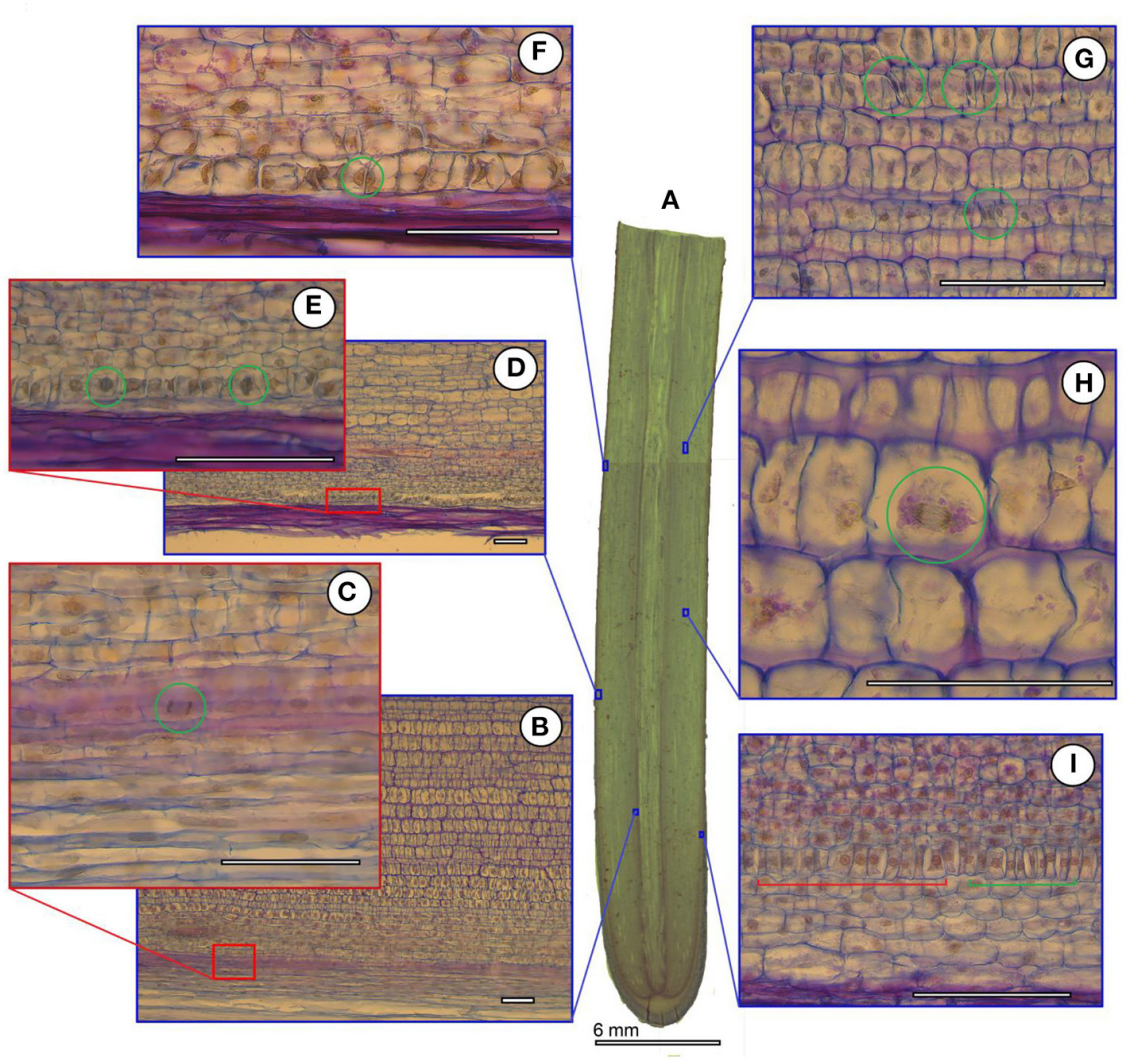
Direct and indirect evidence of continuing cell divisions in a single growth zone on the example of *Monstera deliciosa*: **(A)** general view of a large root at minimum magnification—an accumulation of proliferating cells is visible as dark veins rising along the length of the root along the rhizoderm and pericycle with separate accumulations in the stele and cortex; in the cortex at the pericycle boundary **(B)**, growth and divisions **(C)** occur simultaneously (hereinafter, evidence of divisions is marked by green circles); the rhizoderm **(D)** is characterized by numerous alternating areas of increase due to growth by extension and decrease in length due to synchronous divisions (by **E** in the telophase stage); the same pattern is observed in areas of the rhizoderm much lower along the length of the root **(F)**; in the cortex, areas with longer cells are interspersed with recently divided short cells **(G)**, dividing cells may occur in the anaphase stage **(H)**, in addition, areas with longer cells (marked in red) precede shorter cells (marked in green), which indicates change of the period of growth by periods of divisions **(I)**. Excluding the overview image in all cases, the scale is 100 μm.

There is a lot of interspecific variation in cellular growth patterns among studied aerial roots. In the studied aerial roots, growth at the cellular level occurs differently. The elongation zone lengths varied greatly, but the relative growth rate remained similar, which indicates the same growth pattern. Cells are divided not only in the meristem but also over a significant part of the elongation zone, especially in rhizodermal and pericycle cells (Figure 6). In contrast to most roots of terrestrial plants, there was no sharp increase in the relative growth rate of aerial roots during the transition of meristematic cells to the extension zone. Therefore, throughout this zone, the length of the dividing cells grew in direct proportion to their distance from the root tip, whereas in subterranean roots, the length of the cells grows much faster than their distance from the root tip over the elongation zone. The average relative growth rate of stretching cells in aerial roots was 0.026 h^−1^, which is much lower than the relative growth rate of both stretching and meristematic cells in subterranean roots. It can be assumed that the low relative speed allows the differentiation of stretching cells. Cell growth by stretching lasts more than a week in aerial roots, several times longer than in belowground roots. Thus, at the cellular level, aerial root growth is similar to the growth of stem internodes, leaves of dicotyledons, or succulent fruit, in which divisions can continue during stretching and cells grow at much lower relative rates than in the “typical” roots (Silk, 1984; Shirokova and Ivanov, 1997; Kavanová et al., 2006).

**Figure 7:**
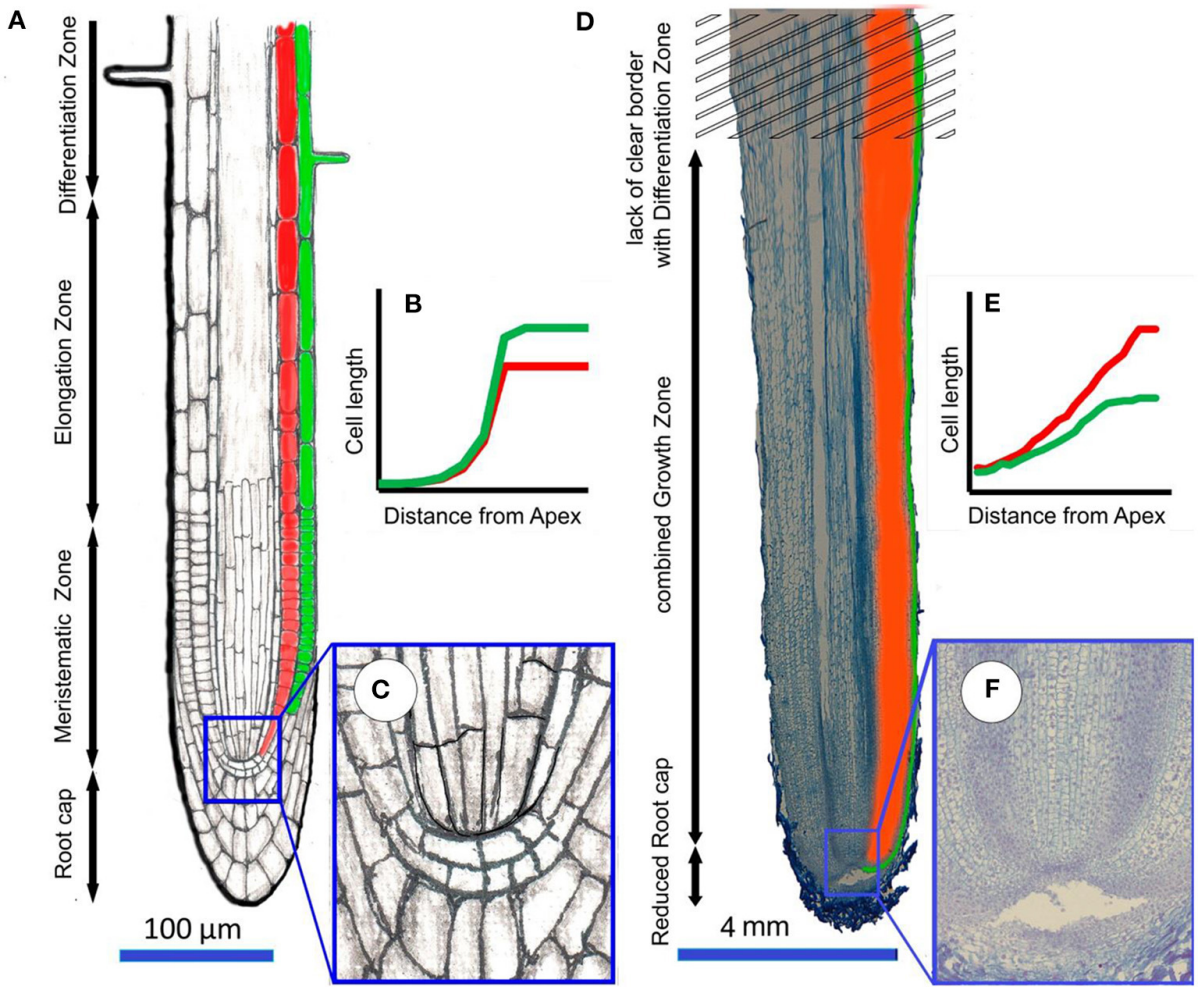
The difference in the organization of the processes of growth of belowground roots (for example *Arabidopsis*) and aerial roots (for example *Cattleya skinneri*). The subterranean root **(A)** is usually relatively thin ( ≤ 1 mm), has a well-developed root cap and clearly localized zones of meristem, elongation and differentiation, which wass diagnosed by the appearance of root hairs; **(B)** the average cell length increases exponentially from meristem to elongation zone, rhizodermal cells are loner than cortex cells; **(C)** detail of closed meristem. In the aerial root **(D)**, which is much thicker than the belowground subterranean root (≥1 mm), the reduced cap, meristem, and elongation zones are merged into a single growth zone, which does not have a clear boundary with the differentiation zone. Root hairs do not develop in the differentiation zone; **(E)** the average cell length increase linearly over a long distance, while cell growth can be accompanied by cell divisions. Cortex cells that have completed growth are longer than rhizodermal cells; **(F)** detail of open meristem.

The low relative growth rate of cortical cells during stretching allows simultaneous divisions in other tissues (rhizodermal) but also, in some cases, among cortex and stele cells. This raises the question of the regulatory mechanisms of such heterogeneous processes as growth and division in one and adjacent tissues. Based on our data, we can identify the following trends characterizing aerial root growth: (1) cortical and rhizodermal cell longitudinal length positively correlates with their distance to the root tip; (2) the length of the extension zone is directly proportional to the growth rate, which is confirmed by direct correlations of the parameters characterizing root growth (Table 3); and (3) the aerial roots of all studied species (except the roots with a reduced meristem) exhibit essentially the same growth pattern, which lacks a sharp increase in relative growth rate during the transition to elongation (Figure 7).

Changes in cortical cell length in the elongation zone of aerial roots differ fundamentally from those of subterranean roots (Figure 7). There, cortical cell length increases sharply in the elongation zone. In aerial roots, cortical cell length increases in direct proportion to the distance from the tip, i.e., there is no sharp increase in the relative growth rate with the transition to extension (Figure 7). A similar phenomenon is observed in the case of metaxylem cells in monocotyledons (not yet studied for cells of other vessels). For example, in corn, onion, and wheat roots, along the meristem, the length of metaxylem cells after the end of mitoses (much closer to the root tip than in other tissues) correlates with their distance to the root tip (Ivanov, 1974). After the beginning of stretching, these cells begin to increase much faster in length. Thus, metaxylem cells no longer divide within the basal two-thirds of the meristem, but they are not stretched, as it was reported before (Dello Ioio et al., 2007); they are growing at a very low relative speed.

With a finite cell length of the cortex and rhizoderm of, on average, 100–150 μm, the root growth rate was equivalent to an increase of <1 cell per row per hour in most species (Table 2). This indicates the presence of a notable feature in slowly growing species (e.g., orchids): mitoses were not visually fixed in sections, which indicates extremely slow mitotic cycles. The conclusion about mitotic activity in the tissues of the zone can be made based on the nuclei morphology. The nuclei of proliferating cells are stained brighter with nucleic dyes and do not have nucleoli. In addition, in some cases, cell lengths decreased, obviously associated with division (Figure 5).

In contrast, in the more rapidly growing *M. deliciosa*, many mitoses are found in the growing zone and rhizoderm, including oblique divisions that lead to polycyte branching. According to our data, mitoses in *M. deliciosa* occurred at a considerable distance and the growth pattern may be intermittent (Eskov et al., 2016). In addition, for some aerial roots with negative geotropism, we described the reduction of the meristem (Eskov et al., 2017). All these indicate the fundamental inapplicability of the RCP method (Ivanov and Dubrovsky, 1997; Zhukovskaya et al., 2018) for the detection of mitotic cycles in aerial roots, which also serves as arguments in favor of a complete difference in the growth pattern of aerial and substrate roots.

A characteristic feature of the aerial roots in the studied species is the presence of large polycytes, which can branch. In *M. deliciosa* roots, the number of polycytes increases with increasing root thickness. It is also assumed that there is a gliding (intrusive) growth in polycytes when proliferating cells and new cell rows can invade intercellular spaces (Eskov et al., 2016). If there exists the gliding type of growth in polycytes, then this is a rather unexpected example of its manifestation because it is traditionally believed that it is peculiar to individual cells: cambial initials, primary and secondary fibers, tracheids, trichosclereids, and some other cells (Snegireva et al., 2010).

We can consider aerial roots with a long elongation zone and higher growth rate, and others with a short elongation zone, and slow growth. Separately, it is necessary to consider aerial roots with deterministic growth, in its extreme manifestation, leading to a reduction in the meristem, which we described in detail previously (Eskov et al., 2017). Using the example of aerial roots of *M. deliciosa*, a special type of root growth organization with a long elongation zone, which is not very similar to the growth of substrate roots, is shown. Duration of cell growth by stretching can last up to a month or more. The distribution of growth along the axis of the aerial root elongation zone is uneven. The contact of neighboring growing polycytes (cell complexes) is accompanied by intrusive growth (Eskov et al., 2016). All plants with long root growth zones that we studied using *M. deliciosa* should have a similar growth type. It can be assumed that a similar root growth type is a characteristic of ecologically similar hemiepiphytes from the aroid family: representatives of the genera *Scindapsus, Rhaphidophora, Philodendron*, and plants of a number of other eco-groups.

The ecological significance of this growth type is that a rather high growth rate is maintained at a considerable distance with high ecophysiological plasticity. In the growth zone, both differentiation and cell division co-occur, photosynthesis is active, and sclereids are present (Gill and Tomlinson, 1971; Benzing, 1996), which enhances the mechanical root strength. Preserving divisions over a vast section, the roots easily overcome the effects of mechanical damage and are rearranged to form subterranean roots when reaching the ground (Gill and Tomlinson, 1977). For another aerial root type with a short elongation zone, a low growth rate is also common. These are, as a rule, typical epiphytes. The ecological role of slow growth, in this case, is a more economical investment of photosynthetic metabolites, greater independence from nitrogen deficiency, and economical use of water.

The fact that plants with long and short elongation zones have the same basic root structure is noteworthy. This is demonstrated in similar relative growth rates and the absence of a pronounced increase in the relative growth rate with the cessation of most mitoses in the cortex. This demonstrates a special type of root growth, quite different from that in substrate roots, but similar to the leaf and stem growth of dicotyledons (Sinnott, 1960). Having revealed a special type of root growth in aerial roots, we assume that the primary reason for such an unusual growth zone organization is the lack of resistance in air, so characteristic of the root as an organ. Thus, in the course of evolution, the aerial root growth became similar to that of other organs that do not experience mechanical resistance.

Roots have evolved over ~400 million years, and if its growth type has not become uncontested, it may be assumed that the basic level of root growth cellular organization is undoubtedly typical (and paleobotanical data most likely confirm this (Hetherington et al., 2016), which can be implemented in two significantly different models. It is too early to suggest that such “dualism” of the root as an organ in general (i.e., all aerial roots are subordinate) or the ability to grow in the air is idiosyncratic to adventive roots (i.e., the primary, main root is not capable of turning into the air in principle). If the second assumption is true, then this can explain the dominance of monocotyledonous plants among epiphytes that do not have a primary root, and among the relatively few dicotyledonous epiphytes, almost complete reduction of the primary root at the very early development stages (e.g., in epiphytic cacti from the Rhipsalideae tribe).

## Conclusions

In the studied aerial roots, there is no clearly defined apical meristem. Instead, there is a single growth zone in which mitotic divisions in the cortical parenchyma and rhizoderm continue over a significant part of the elongation zone. The length of the elongation zone is directly proportional to the growth rate. Thus, the relative rate shows relatively less variation, in spite of differences in ecomorphology and taxonomy.

## Data Availability Statement

The original contributions presented in the study are included in the article/supplementary material, further inquiries can be directed to the corresponding author.

## Author Contributions

AE conducted data curation and wrote the original draft. GZ and AE contributed to conceptualization. EA acquired funding. VV and AE assisted with formal analysis. AE, GZ, and EA reviewed and edited the manuscript. All authors have read and agreed to the published version of the manuscript.

## Funding

This work was supported by the Federal budget of Russian Federation, Grant to support for the creation and development of a World-Class Scientific Center Agrotechnologies for the Future, Project No. 075-15-2022-322 date 22.04.2022 and accordance with Institutional Research Project No. 122042700002-6 at the Unique Scientific Installation Fund Greenhouse. Also, the work was supported by MSU Contract #121032500089-1. In this work we used a microscope purchased under the program of modernization of scientific equipment of St. Petersburg State University No. 1.40.541.2017.

## Conflict of Interest

The authors declare that the research was conducted in the absence of any commercial or financial relationships that could be construed as a potential conflict of interest.

## Publisher's Note

All claims expressed in this article are solely those of the authors and do not necessarily represent those of their affiliated organizations, or those of the publisher, the editors and the reviewers. Any product that may be evaluated in this article, or claim that may be made by its manufacturer, is not guaranteed or endorsed by the publisher.

## Supplementary Material

Supplementary Table S1 Primary data of growth rate determinations of aerial roots of 20 species.

Supplementary Table S2 Primary data of cell length measurements from anatomical sections of aerial roots of 12 out of 20 species.
